# First Observation of Hemoglobin Kansas [β102(G4)Asn→Thr, AAC>ACC] in the Turkish Population

**DOI:** 10.4274/tjh.2015.0177

**Published:** 2015-12-03

**Authors:** İbrahim Keser, Alev Öztaş, Türker Bilgen, Duran Canatan

**Affiliations:** 1 Akdeniz University Faculty of Medicine, Department of Biology and Genetics, Antalya, Turkey; 2 Melid Park Private Hospital, Malatya, Turkey; 3 Research and Application Center for Scientific and Technological Investigations (NABİLTEM) of Namık Kemal University, Tekirdağ, Turkey; 4 Antalya Diagnostic Center of Genetic Diseases, Antalya, Turkey

**Keywords:** Abnormal hemoglobins, Hb Kansas, Turkish population

## TO THE EDITOR

Hemoglobin (Hb) Kansas [β102 (G4) Asn→Tyr, AAC>ACC] is an unstable abnormal hemoglobin with low oxygen affinity and increased dissociation. Hb Kansas has rarely been reported in the literature to date; the first case was defined in the state of Kansas of the United States [1]. The second reported case was a newborn baby with cyanosis from Sarajevo and the third was an elderly patient with polycythemia from Japan [2,3]. There has been no previous report from Turkey [4]. We herein report the first case of Hb Kansas from Turkey, an introduction of clinical significance.

**Figure 1:**
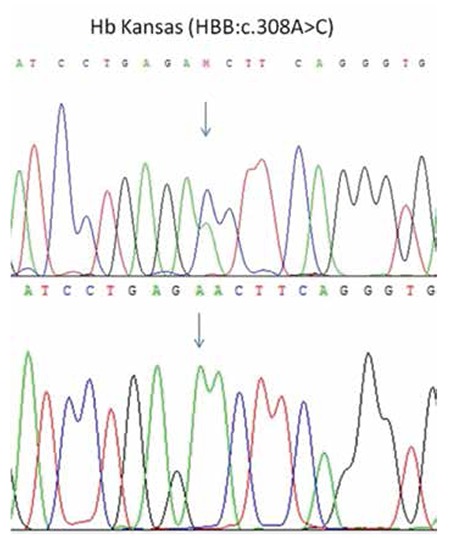
Hemoglobin Kansas in DNA sequencing.

Case: A 28-year-old male patient with cyanosis of the lips and fingertips was admitted to a hospital in the city of Malatya. He had peripheral cyanosis of the hands and feet on physical examination. Blood gas analysis showed low oxygen levels. Complete blood count, blood chemistry, and cardiac echocardiography results were within normal levels. High-performance liquid chromatography results were as follows; HbA1: 63.6%, HbA2: 32.8%, HbF: 0.2%. Agarose gel electrophoresis was performed to distinguish HbA2, but a band was identified at the level of 39.7% in HbF, G zone, and between HbA1 and HbA2. A blood sample was transferred to our genetic diagnostic center. Following DNA extraction with a commercial kit (Roche, Germany) and amplification of the whole beta globin gene by standard PCR protocols, DNA sequencing (Applied Biosystems, USA) revealed an A to C substitution at nucleotide position 308 (Figure 1). This change was identified as HBB: c.308 A>C, known as Hb Kansas in the HbVar database [5].

Hb Kansas is one of four known hemoglobins with neutral substitutions, along with Hb Köln, Porto Alegre, and Genova [1].

The oxygen equilibrium of Hb Kansas has two unusual characteristics: low affinity for oxygen and low heme-heme interaction. The low oxygen affinity of Hb Kansas should be considered in the differential diagnosis of peripheral cyanosis, especially in the neonatal period and in cyanotic disease and polycythemia in the elderly.
